# Factors Associated With Unplanned 30-Day Readmissions After Hematopoietic Cell Transplantation Among US Hospitals

**DOI:** 10.1001/jamanetworkopen.2019.6476

**Published:** 2019-07-05

**Authors:** Binod Dhakal, Smith Giri, Adam Levin, Lisa Rein, Timothy S. Fenske, Saurabh Chhabra, Nirav N. Shah, Aniko Szabo, Anita D’Souza, Marcelo Pasquini, Parameswaran Hari, Mehdi Hamadani

**Affiliations:** 1 Division of Hematology and Oncology, Medical College of Wisconsin, Milwaukee; 2 Division of Hematology and Oncology, Yale University, New Haven, Connecticut; 3 Division of Biostatistics, Medical College of Wisconsin, Milwaukee

## Abstract

**Importance:**

Hematopoietic cell transplantation (HCT) is a therapeutic strategy in the management of several hematological cancers. Limited data exist on the incidence and predictors of 30-day readmission after HCT.

**Objective:**

To measure the incidence of and risk factors associated with 30-day readmission following HCT in the United States.

**Design, Setting, and Participants:**

This cohort study examined patient data from the US population-based Nationwide Readmissions Database. All adults (age ≥18 years) who underwent autologous (auto-) or allogenic (allo-) HCT in US hospitals between January 1, 2012, and November 30, 2014, were included. The analysis was performed from June 2018 to February 2019.

**Main Outcomes and Measures:**

The main outcome was 30-day readmission rates for auto-HCT and allo-HCT. Factors associated with readmission, including baseline demographic characteristics and disease- and hospital-related characteristics (including annual case volume), were measured.

**Results:**

A total of 28 356 index admissions for auto-HCT in 244 centers (191 low-volume, 38 medium-volume, and 15 high-volume centers) and 17 217 index admissions for allo-HCT in 211 centers (161 low-volume, 37 medium-volume, and 13 high-volume centers) were identified during the study period. The overall 30-day readmission rates were 11.6% for auto-HCT and 24.4% for allo-HCT. The odds of readmission were significantly higher in low-volume hospitals compared with high-volume hospitals (adjusted odds ratio [aOR], 1.69; 95% CI, 1.08-2.64 for auto-HCT and aOR, 1.41; 95% CI, 1.09-1.82 for allo-HCT) but comparable to medium-volume hospitals (aOR, 1.06; 95% CI, 0.62-1.83 for auto-HCT and aOR, 1.19; 95% CI, 0.90-1.57 for allo-HCT). Other factors associated with readmission for auto-HCT included younger age (aOR for age ≥50 vs <49 years, 0.82; 95% CI, 0.68-0.98), female sex (aOR, 1.21; 95% CI, 1.06-1.36), disease type (aOR for other vs myeloma, 1.37; 95% CI, 1.06-1.77), and Elixhauser comorbidity index score (aOR for ≥20 vs 0, 1.5; 95% CI, 1.17-1.93). For allo-HCT, factors associated with readmission included disease type (aOR for acute lymphoblastic leukemia vs acute myelogenous leukemia, 1.30; 95% CI, 1.04-1.62), insurance (aOR for Medicare vs private, 1.18; 95% CI, 1.02-1.36), and Elixhauser comorbidity index score (aOR for 1-9 vs 0, 1.2; 95% CI, 1.04-1.39). Infections, neutropenic fever, and gastrointestinal symptoms were the most common reasons for readmission for both types of HCT.

**Conclusions and Relevance:**

This study found substantial rates of readmission for both types of HCT and an inverse association between hospital HCT volume and 30-day readmission. These results may provide guidance when developing quality indicators and policies penalizing hospitals for HCT readmission.

## Key Points

**Question:**

What is the risk of readmission among adults receiving hematopoietic cell transplants in the United States?

**Findings:**

This cohort study of a US population-based database found that 30-day readmission rates for autologous and allogenic hematopoietic cell transplants were 11.6% and 24.4%, respectively. The most common reasons for readmission included infection and gastrointestinal symptoms, and hospital volume was inversely associated with readmission rate.

**Meaning:**

This study suggests that a substantial proportion of US adults receiving hematopoietic cell transplants are readmitted within 30 days of discharge, which may lead to significant health care expenditures.

## Teaser

This cohort study estimates the risk of 30-day hospital readmission among adults receiving autologous and allogenic hematopoietic cell transplants in the United States.

## Introduction

Autologous (auto-) and allogeneic (allo-) hematopoietic cell transplantation (HCT) are widely used therapies in the management of several hematological disorders.^1^ Despite its curative potential, HCT is often associated with significant treatment-related morbidity, mortality, and health care utilization. A 2009 report from the Agency for the Healthcare Research and Quality (AHRQ) for the Healthcare Cost and Utilization Project (HCUP)^2^ noted that HCT was among the 10 procedures with the greatest increase in hospital costs from 2004 to 2007, with a growth rate of 84.9% from $694 million to $1.3 billion.

Readmission after index hospitalization is a commonly used quality and cost-containment metric. Under the Hospital Readmissions Reduction Program, the Centers for Medicare & Medicaid Services uses 30-day unplanned readmission rate as a quality metric to penalize hospitals with high rates of risk-adjusted readmissions for medical conditions such as heart failure, pneumonia, myocardial infarction, and conditions requiring surgery.^3,4,5,6^ With increasing health care demands and cost of care for patients with cancer, the Centers for Medicare & Medicaid Services introduced the Oncology Care Model in 2015. This high-value care model aims to provide quality care at the same or lower price.^7^ To assess cancer care performance in the model, one of the quality metrics proposed is all-cause unplanned readmission. In this context, patients undergoing auto-HCT and allo-HCT are particularly vulnerable to unplanned readmissions due to immune suppression, myelosuppression, graft-vs-host disease (GVHD), and infections. To our knowledge, data on readmission rates and their associated factors in patients undergoing HCT are lacking. We designed this comprehensive study to assess the incidence and associated risk factors of unplanned 30-day readmission after auto-HCT and allo-HCT.

## Methods

### Data Source

The study used data from the Nationwide Readmissions Database (NRD), part of a family of databases developed by AHRQ, from January 1, 2012, to November 30, 2014. The analysis was performed from June 2018 to February 2019. The NRD is a unique and powerful database of hospital inpatient stays for patients of all ages and payers. It is drawn from AHRQ’s state inpatient databases, which have a variety of sizes and densities.^8^ The database contains approximately 17 million discharges every year, weighted to roughly 36 million discharges nationwide. The NRD contains clinical and nonclinical variables that support readmission analyses within the same state and calendar year. The study was deemed exempt by the Medical College of Wisconsin institutional review board because the NRD is publicly available and contains deidentified data. This study follows the Strengthening the Reporting of Observational Studies in Epidemiology (STROBE) reporting guideline.^9^

### Study Population

We identified the index admission for HCT using *International Classification of Diseases, Ninth Revision* (*ICD-9*) procedure codes (auto-HCT: 41.00, 41.01, 41.04, 41.07, and 41.09; allo-HCT: 41.02, 41.03, 41.05, 41.06, and 41.08) (eTable 1 in the Supplement). Survey-weighted domain analysis was conducted to study incidence of 30-day readmission by center volume. Annual hospital case volume was calculated as the sum of all discharges with HCT within the calendar year; groups with low, medium, and high annual case volume were created based on (survey-weighted) tertiles of patients in the analytic data domain (eFigure in the Supplement). Accordingly, hospitals were classified as low volume (<58 per year), medium volume (58-157 per year), and high volume (≥158 per year) for allo-HCT and low volume (<78 per year), medium volume (78-186 per year), and high volume (≥187 per year) for auto-HCT. The Centers for Medicare & Medicaid Services does not provide hospital classification as low-, medium-, and high-volume hospitals for HCT. Patients discharged in the month of December were excluded from the study (to allow 30-day readmission window in the full calendar year of data), as the primary outcome of the study was 30-day readmission. The analysis was limited to urban teaching hospitals because there were few eligible hospitalizations in rural and nonteaching hospitals. Index admissions for patients who died or transferred to another acute care hospital were excluded.

### Main Outcome Measure

The main outcome measure was 30-day, unplanned, all-cause hospital readmission following an index admission for HCT. Readmissions were identified according to the methods outlined by the Centers for Medicare & Medicaid Services.^10^ For patients who had multiple readmissions within the first 30 days, only the first readmission was included. To determine the cause of readmission, the primary diagnosis of each readmission was independently reviewed by 2 of us (B.D. and S.G.) and grouped into clinically relevant categories. Secondary outcomes examined were length of stay (LOS) and hospital costs of readmission. Cost data are not directly available in the NRD; all costs were estimated from total charges using annual hospital-specific cost-to-charge ratios provided by HCUP. The estimated costs were then adjusted to the national mean hospital wage using the area wage index provided by HCUP.

### Index Admission Demographic and Clinical Characteristics

We included patient demographic characteristics (age, sex, race, expected primary payer, and median household income) and relevant comorbidities. We used *ICD-9* codes to determine the underlying disease for which HCT was performed. Hospital characteristics, such as volume and bed size, were also examined. The HCUP Clinical Classification Software and *ICD-9 Clinical Modification* codes used to define these variables are listed in eTable 1 in the Supplement.

### Statistical Analysis

We used weights developed by AHRQ to estimate national index admissions for HCT throughout the United States.^8^ After the weighting, we summarized the patient demographic and clinical characteristics. We presented categorical variables with frequencies and percentages and continuous variables with means (standard deviations) and medians (interquartile ranges [IQRs]), as appropriate. Patient demographic characteristics, comorbidities, and hospital characteristics between low-, medium-, and high-volume hospitals were compared using the χ^2^ test for categorical variables and 1-way analysis of variance for continuous variables; nonnormally distributed continuous variables were log-transformed prior to analysis of variance. To examine differences in 30-day readmissions among low–, medium–, and high–HCT volume hospitals, survey-weighted multivariable logistic regression models were constructed. The model was used to compare odds of readmission between center volumes after controlling for potential confounders, including age, sex, insurance, median income (by zip code), hospital bed size, Elixhauser comorbidity index (ECI) for readmission (eTable 2 in the Supplement), disease type, presence of acute GVHD (aGVHD) for allo-HCT only, use of total body irradiation, stem cell source, discharge disposition, presence of infection, and LOS during index admission. We used adjusted odds ratios (aORs) and 95% confidence intervals to report the results of regression analysis. To address the issues of patients readmitted to a center in a different state than the one in which HCT was performed, we performed analysis for state residents only using the HCUP data element RESIDENT.

All analyses were completed using SAS statistical software version 9.4 (SAS Institute Inc). All statistical tests were performed using SAS survey procedures to account for the NRD sampling design per HCUP guidelines. All *P* values were 2-sided and *P* < .05 was considered statistically significant.

## Results

**Figure 1.**
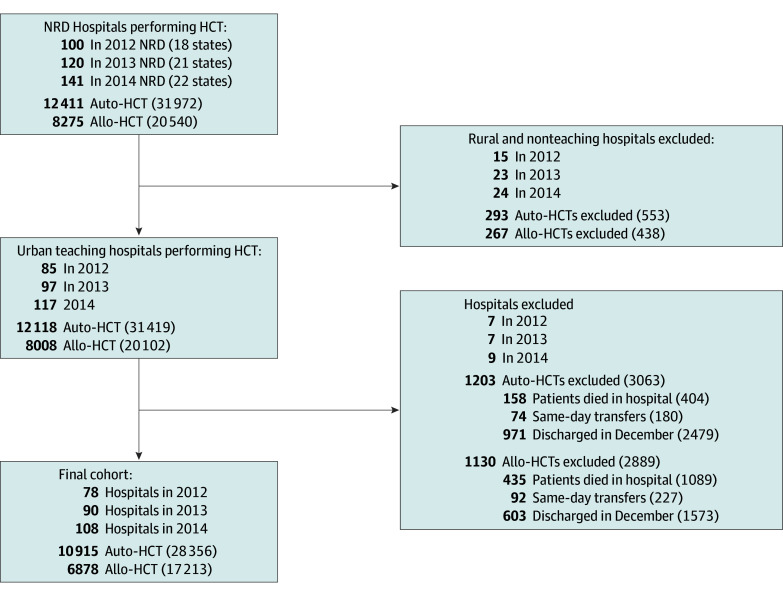
Study Population and Selection Criteria for Autologous (Auto-) and Allogeneic (Allo-) Hematopoietic Cell Transplants (HCT) Numbers in parentheses are weighted values. NRD indicates Nationwide Readmissions Database.

Between January 1, 2012, and November 30, 2014, there were 28 356 index admissions for auto-HCT in 244 centers and 17 217 index admissions for allo-HCT in 211 centers (Figure 1). For auto-HCT, 191 hospital-years (78%) were categorized as low–, 38 (16%) as medium–, and 15 (6%) as high–transplant volume centers, while for allo-HCT, 161 (76%) were classified as low–, 37 (18%) as medium–, and 13 (6%) as high–transplant volume centers.

### Baseline Characteristics

Table 1 summarizes the patient and hospital characteristics for auto- and allo-HCT between low–, medium–, and high–HCT volume hospitals for patients who survived to discharge. Acute myelogenous leukemia was the most common indication for allo-HCT, while multiple myeloma was the most common indication for auto-HCT. These characteristics were well matched among the 3 transplant volume categories for both modalities. There was a significant difference in the LOS at index admission for auto-HCT, with longer LOS in low– and medium–transplant volume hospitals compared with high–transplant volume hospitals. The distribution of comorbidities was well matched between the groups, as shown by the ECI score in Table 1.

**Table 1.** Baseline Patient and Hospital Characteristics for Index Transplantation Admission, 2012 to 2014

Variable	Autologous HCT (n = 28 356)				Allogeneic HCT (n = 17213)			
	Low (n = 9290)	Medium (n = 9345)	High (n = 9721)	*P* Value	Low (n = 5483)	Medium (n = 5743)	High (n = 5987)	*P* Value
Hospital-years, No.	192	38	15		161	37	13	
Patients aged ≥50 y, No. (%)	7041 (76)	7193 (77)	7455 (77)	.82	3187 (58)	3578 (62)	3909 (65)	.009
Male, No. (%)	5666 (61)	5573 (60)	5980 (62)	.32	3250 (59)	3280 (57)	3506 (59)	.57
Primary insurance payer, No. (%)								
Medicare	2869 (31)	2565 (27)	2575 (26)	.12	1047 (19)	1208 (21)	1165 (19)	.08
Medicaid	1167 (13)	1016 (11)	847 (9)		820 (15)	562 (10)	569 (10)	
Private	4712 (51)	5186 (56)	5871 (60)		3232 (59)	3560 (62)	3949 (66)	
Self-pay	542 (6)	578 (6)	429 (4)		384 (7)	413 (7)	305 (5)	
Zip code income, median quartile, No. (%)								
Lowest	1851 (20)	1703 (18)	2173 (22)	.33	956 (17)	817 (14)	1208 (20)	.51
Low-medium	2234 (24)	2658 (28)	2341 (24)		1381 (25)	1378 (24)	1314 (22)	
Medium-high	2377 (26)	2501 (27)	2345 (24)		1470 (27)	1593 (28)	1496 (25)	
Highest	2638 (28)	2389 (26)	2659 (27)		1579 (29)	1867 (33)	1814 (30)	
Unknown	189 (2)	95 (1)	202 (2)		97 (2)	87 (2)	155 (3)	
Diagnosis, No. (%)								
Multiple myeloma	5262 (57)	5810 (62)	5889 (61)	.09	814 (15)^a^	823 (14)^a^	1129 (19)^a^	<.001
Non-Hodgkin lymphoma	1996 (22)	1699 (18)	1940 (20)					
Hodgkin lymphoma	725 (8)	706 (8)	754 (8)					
Acute myelogenous leukemia	NA	NA	NA		2606 (48)	2682 (47)	2728 (46)	
Acute lymphoblastic leukemia	NA	NA	NA		821 (15)	875 (15)	944 (16)	
Myelodysplastic syndrome	NA	NA	NA		654 (12)	881 (15)	804 (13)	
Other	1307 (14)	1130 (12)	1138 (12)		589 (11)	481 (8)	382 (6)	
Stem cell source, No. (%)								
Peripheral blood	8832 (95)	8963 (96)	9205 (95)	.64	4626 (84)	4962 (86)	5259 (88)	.81
Bone marrow	458 (5)	383 (4)	517 (5)		632 (12)	567 (10)	529 (9)	
Cord blood	NA	NA	NA		225 (4)	213 (4)	199 (3)	
Total body irradiation, No. (%)	77 (0.8)	42 (0.4)	53 (0.5)	.30	584 (11)	956 (17)	1265 (21)	.01
Nonroutine discharge disposition, No. (%)	1497 (16)	1396 (15)	1328 (14)	.63	1584 (29)	1874 (33)	1359 (23)	.34
Infection index admission, No. (%)	3192 (34)	3350 (36)	4087 (42)	.04	2583 (47)	2649 (46)	3246 (54)	.03
Acute graft-vs-host disease index admission, No. (%)	54 (0.6)	135 (1.5)	23 (0.2)	<.001	922 (17)	663 (12)	700 (12)	.06
Elixhauser comorbidity score for readmission, No. (%)								
0	2408 (26)	2196 (24)	2258 (23)	.35	1577 (29)	1626 (28)	1864 (31)	.75
1-9	2668 (29)	3082 (33)	3039 (31)		1826 (33)	1956 (34)	1821 (30)	
10-19	2187 (24)	2264 (24)	2492 (26)		1285 (23)	1374 (24)	1495 (25)	
≥20	2027 (22)	1803 (19)	1933 (20)		795 (14)	787 (14)	807 (13)	
Large hospital bed size, No. (%)	6759 (73)	8111 (87)	8138 (84)	.37	3706 (68)	5039 (88)	4664 (78)	.30
Length of stay, median (IQR), d	17.6 (14.5-20.5)	17.6 (15.1-21.0)	17.5 (15.3-20.3)	.02	25.7 (21.0-32.3)	24.4 (20.5-29.8)	24.6 (21.6-30.4)	.17

Abbreviations: HCT, hematopoietic cell transplant; IQR, interquartile range; NA, not applicable.

^a^ For allogenic HCT, multiple myeloma, non-Hodgkin lymphoma, and Hodgkin lymphoma are combined.

### 30-Day Readmissions After HCT

**Figure 2.**
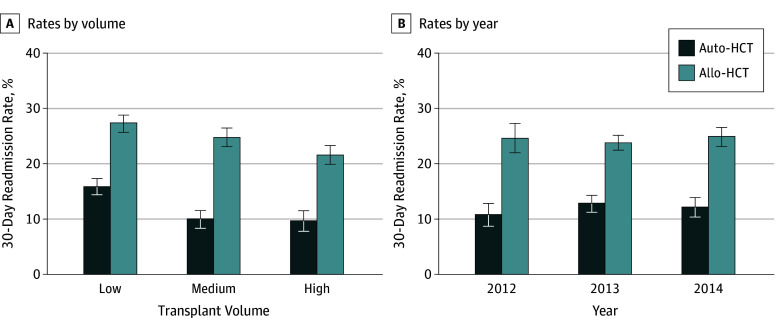
Readmission Rates by Transplant Volume and Year A, Readmission rates in low-, medium-, and high-volume centers for autologous (auto-) and allogenic (allo-) hematopoietic cell transplant (HCT). B, Readmission rates in 2012 to 2014 for auto-HCT and allo-HCT. Error bars indicate standard errors.

The overall incidence of 30-day readmission was 11.6% for auto-HCT and 24.4% for allo-HCT (eTable 3 in the Supplement). High-volume hospitals had significantly lower rates of readmission compared with low-volume hospitals (high vs medium vs low, 9.6% vs 9.5% vs 15.8% for auto-HCT and 21.5% vs 24.7% vs 27.2% for allo-HCT) (Figure 2A; eTable 3 in the Supplement). When including state residents only, readmission rates for low vs medium vs high volume were 16.4% vs 10.5% vs 10.4% for auto-HCT and 26.8% vs 25.1% vs 21.3% for allo-HCT. There was no difference in the readmission rates by year of transplant for both HCT modalities (Figure 2B). eTable 3 in the Supplement summarizes the univariate analysis of patients readmitted within 30 days. The rates of infection and mean LOS at index admission were significantly lower for readmitted patients for auto-HCT, while there was no difference in rates of infection, LOS, and aGVHD at index admission for patients undergoing allo-HCT.

The readmission characteristics are summarized in eTable 4 in the Supplement. Most readmissions were to the hospital in which the transplant was performed. The median (range) time to readmission was 5.5 (2.9-11.3) days for auto-HCT and 9.3 (4.5-16.5) days for allo-HCT.

On adjusted analysis, center volume remained independently associated with 30-day readmission after HCT (Table 2). Odds of readmission were significantly higher in low-volume hospitals compared with high-volume hospitals (aOR, 1.69; 95% CI, 1.08-2.64 for auto-HCT and aOR, 1.41; 95% CI, 1.09-1.82 for allo-HCT) but comparable to medium-volume hospitals (aOR, 1.06; 95% CI, 0.62-1.83 for auto-HCT and aOR, 1.19; 95% CI, 0.90-1.57 for allo-HCT). As shown in Table 2, other factors associated with higher 30-day readmission for auto-HCT included age (aOR for age ≥50 vs <49 years, 0.82; 95% CI, 0.68-0.98), female sex (aOR, 1.21; 95% CI, 1.06-1.36), higher ECI (aOR for ≥20 vs 0, 1.5; 95% CI, 1.17-1.93), disease type (aOR for other vs myeloma, 1.37; 95% CI, 1.06-1.77), and nonroutine discharge (discharge to skilled nursing facility, short-term hospital, or home health care). For allo-HCT, on the other hand, the associated factors were hospital bed size (aOR, 0.75; 95% CI, 0.57-0.95 for small-size hospitals), higher ECI (aOR for 1-9 vs 0, 1.2; 95% CI, 1.04-1.39), diagnosis with acute lymphoblastic leukemia (aOR for acute lymphoblastic leukemia vs acute myelogenous leukemia, 1.30; 95% CI, 1.04-1.62), insurance (aOR for Medicare vs private, 1.18; 95% CI, 1.02-1.36), and use of cord blood transplants (aOR, 2.40; 95% CI, 1.83-3.16). Length of stay at index admission was inversely associated with readmissions only for auto-HCT (aOR, 0.94; 95% CI, 0.90-0.95).

**Table 2.** Multivariable Analysis of Variables Associated With 30-Day Unplanned Readmissions

Variable	Autologous HCT		Allogenic HCT	
	aOR (95% CI)	*P* Value	aOR (95% CI)	*P* Value
Hospital case volume				
High	1 [Reference]		1 [Reference]	
Medium	1.06 (0.62-1.83)	.83	1.19 (0.90-1.57)	.22
Low	1.69 (1.08-2.64)	.02	1.41 (1.09-1.82)	.009
Age, y				
18-49	1 [Reference]	.03	1 [Reference]	.24
≥50	0.82 (0.68-0.98)		0.90 (0.77-1.07)	
Sex				
Male	1 [Reference]	.003	1 [Reference]	.74
Female	1.21 (1.07-1.36)		1.02 (0.89-1.17)	
Zip code income, median quartile				
Lowest	1 [Reference]		1 [Reference]	
Low-medium	0.83 (0.66-1.04)	.11	0.94 (0.76-1.18)	.60
Medium-high	0.79 (0.62-1.00)	.05	0.81 (0.66-0.99)	.04
Highest	0.80 (0.63-1.01)	.06	0.90 (0.72-1.12)	.34
Unknown	1.06 (0.70-1.62)	.77	0.73 (0.47-1.14)	.16
Hospital bed size				
Large	1 [Reference]	.15	1 [Reference]	.04
Small or medium	0.72 (0.47-1.12)		0.75 (0.57-0.98)	
Elixhauser comorbidity score for readmission				
0	1 [Reference]		1 [Reference]	
1-9	1.13 (0.90-1.40)	.29	1.20 (1.04-1.39)	.01
10-19	1.03 (0.81-1.32)	.79	1.45 (1.21-1.73)	<.001
≥20	1.50 (1.17-1.93)	.002	1.36 (1.10-1.69)	.005
Diagnosis				
Multiple myeloma	1 [Reference]		NA	
Non-Hodgkin lymphoma	1.27 (0.98-1.63)	.06	NA	
Hodgkin lymphoma	0.91 (0.66-1.25)	.56	NA	
Others including acute myelogenous leukemia	1.37 (1.06-1.77)	.02	NA	
Acute myelogenous leukemia	NA		1 [Reference]	
Acute lymphoblastic leukemia	NA		1.30 (1.04-1.62)	.02
Myelodysplastic syndrome	NA		0.97 (0.78-1.21)	.78
Hodgkin lymphoma, non-Hodgkin lymphoma, and multiple myeloma	NA		1.11 (0.93-1.31)	.25
Other	NA		0.91 (0.72-1.15)	.42
Stem cell source (allogenic HCT only)				
Peripheral blood	NA		1 [Reference]	
Bone marrow	NA		1.03 (0.82-1.30)	.80
Cord blood	NA		2.40 (1.83-3.16)	<.001
Insurance				
Private or health maintenance organization	1 [Reference]		1 [Reference]	
Medicare	1.06 (0.90-1.25)	.47	1.18 (1.02-1.36)	.03
Medicaid	1.13 (0.89-1.45)	.32	1.16 (0.89-1.52)	.27
Self-pay, other, or missing	0.65 (0.48-0.86)	.003	1.28 (1.03-1.60)	.03
Discharge disposition				
Routine	1 [Reference]	.005	1 [Reference]	.82
Nonroutine	1.39 (1.10-1.74)		0.98 (0.86-1.12)	
Index hospitalization length of stay, d				
Continuous, units of 1 d	0.94 (0.90-0.99)	.01	1.00 (1.00-1.00)	.88
Infection				
No infection	1 [Reference]	.16	1 [Reference]	.12
Infection	0.88 (0.73-1.05)		1.11 (0.97-1.26)	
Graft-vs-host disease (allogenic HCT only)				
No	NA		1 [Reference]	.26
Yes	NA		1.12 (0.92-1.36)	
Total body irradiation (allogenic HCT only)				
No	NA		1 [Reference]	.21
Yes	NA		1.13 (0.93-1.38)	

Abbreviations: aOR, adjusted odds ratio; HCT, hematopoietic cell transplant; NA, not applicable.

### LOS and Cost of 30-Day Readmissions

There was a significant difference in readmission LOS among readmitted patients from low-, medium-, and high-volume index facilities (median [IQR], 6.9 [3.2-15.2] days, 5.4 [2.7-12.6] days, and 5.3 [2.7-11.7] days; *P* = .03) for allo-HCT but not for auto-HCT (median [IQR], 5.1 [2.7-8.5] days, 4.7 [2.4-10.5] days, and 4.2 [2.3-7.6] days; *P* = .24) (eTable 4 in the Supplement). The readmission cost was significantly higher in low-volume hospitals compared with medium- and high-volume hospitals (mean [SE], $19 794 [$1221], $20 968 [$1811], and $17 294 [$2019]; *P* < .001 for auto-HCT and $47 823 [$3215], $43 731 [$4028], and $35 889 [$3624]; *P* < .001 for allo-HCT). However, there was no significant difference in readmission costs by index facility volume for auto-HCT or allo-HCT (eTable 4 in the Supplement).

### Reasons for 30-Day Readmissions

**Figure 3.**
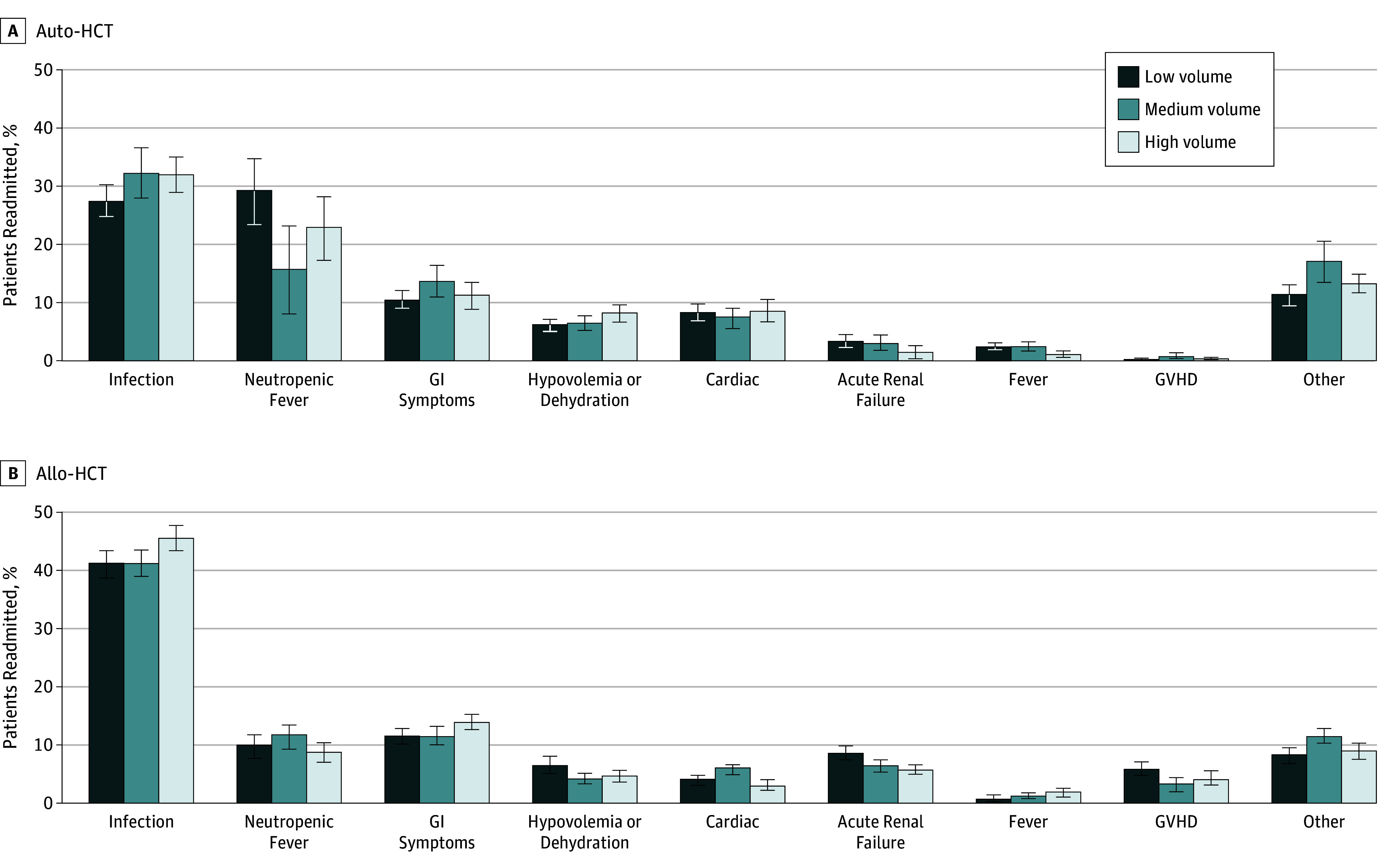
Reasons for 30-Day Readmission After Hematopoietic Cell Transplantation (HCT) A, Readmission reasons after autologous HCT (auto-HCT) by hospital transplant volume. B, Readmission reasons after allogenic HCT (allo-HCT) by hospital transplant volume. Error bars indicate standard errors; GI, gastrointestinal; and GVHD, graft-vs-host disease.

For auto-HCT, the most common reasons for readmission included infections (30%), neutropenic fever (24%), gastrointestinal (GI) symptoms (12%), and other causes (14%). For allo-HCT, infections remained the most common reason (43%), followed by GI symptoms (12%) and neutropenic fever (10%). Graft-vs-host disease constituted 5% of readmission after allo-HCT. Readmission reasons by transplant volume for both modalities are shown in Figure 3. In addition, our study found several other important reasons for readmission. Cardiac reasons were present in 8.3% for allo-HCT compared with 4.7% for auto-HCT. Acute renal failure was present in 7% of allo-HCT vs approximately 3% of auto-HCT. Dehydration leading to hypovolemia as the reason for readmission was comparable (7% in auto- vs 5% in allo-HCT). Fever without any specific diagnosis was also comparable, occurring in 1.7% and 2.1% of patients who underwent allo-HCT and auto-HCT, respectively.

## Discussion

Using a large national readmission database, we report the incidence of and factors associated with unplanned 30-day readmission after HCT in the United States. The overall incidence of readmission was 11.6% for auto-HCT and 24.4% for allo-HCT. We report an inverse association between hospital transplant volume and 30-day readmissions in addition to several novel associations. Infections and neutropenic fever were the most common reasons for readmission.

Patients with cancer generally have higher rates of 30-day unplanned readmissions (approximately 25%) compared with patients with other conditions.^11,12^ There is a paucity of data on readmissions in hematological malignant neoplasms, particularly for patients undergoing HCT. The reported rates of unplanned readmission in published studies, mainly comprising patients undergoing allo-HCT, vary from 20% to 58%.^13,14,15,16^ This wide range is partly due to variance in the time frame used for reporting readmission rates in HCT, depending on the study. We used a 30-day readmission rate, a time frame widely accepted as reasonable for assessing quality of care received during the preceding hospitalization.^17^ Our results showed that the readmission rates in allo-HCT were comparable to readmission among other patients with cancer, while rates were much lower after auto-HCT.

The association between high volume and superior clinical outcomes has been replicated in several studies in the setting of HCT (mainly allo-HCT), although a threshold for what is considered high HCT volume has not been consistently defined.^18,19,20^ The clinical outcomes studied with HCT volume include transplant-related mortality, survival, and treatment failure. To our knowledge, no studies have investigated relapse and readmission rates, and this study is the first to report an association between HCT volume and readmission rates. In the absence of a universally accepted definition of high HCT volume, our annual case volume groups were created based on (survey-weighted) tertiles of patients in the analytic data domain. For both auto-HCT and allo-HCT, our results showed that readmission rates were significantly lower in high-volume and medium-volume hospitals compared with low-volume hospitals. For other characteristics of hospitals potentially associated with rates of readmission, we found no association between hospital bed size and auto-HCT readmission, but significantly lower readmission for larger hospitals for allo-HCT. Possible drivers of the observed inverse association between hospital transplant volume and 30-day readmissions are better patient selection, proficiency in HCT procedures by more experienced clinicians and ancillary staff, and better care coordination after HCT at high-volume centers. Several of these factors may affect LOS during readmission and, consequently, the lower cost observed in high-volume centers.

Published studies have identified several risk factors associated with 30-day unplanned readmission after HCT. These include use of myeloablative or total body irradiation regimens, infections or GVHD during the index HCT admission, higher HCT comorbidity index, Medicaid coverage, and low median income for patients undergoing allo-HCT.^13,15,21,22,23^ In addition, patients undergoing cord blood transplants and patients with acute lymphoblastic leukemia were also at high risk for readmission.^24,25^ Of these, our study identified 3 factors in addition to center volume that were associated with readmission after allo-HCT: higher ECI, cord blood transplantation, and diagnosis of acute lymphoblastic leukemia. In addition, our study did not find an association between income and readmission risk. Several associations were identified for auto-HCT, but 2 worth highlighting are LOS at index admission and nonroutine discharge. In contrast to a previous study,^16^ we found an inverse association between the LOS at index admission and readmission rates. Of note, a recent study in patients with heart failure also showed an inverse association of LOS with readmission rates, likely due to hospitals’ early discharge policies.^26^ Discharge before the resolution of complications following transplant could result in readmission in patients who have undergone HCT, but the divergent results observed between auto-HCT and allo-HCT preclude more definite conclusions. The mean LOS for auto-HCT was approximately 17 days compared with 25 days for allo-HCT. Patients discharged to short-term hospitals, skilled nursing facilities, and home health care were more likely to be readmitted, indicating that this population may not be fully recovered following transplant. The association, however, was not observed in patients who underwent allo-HCT.

Previous studies^13,14,15,16,21,22^ have reported fevers with and without infections, GI toxic effects, and graft complications (mainly GVHD) as some of the common causes of readmission. These results were corroborated by our study as the most common cause of readmission for both types of HCT was infection, followed by neutropenic fever and GI toxic effects. After infections, GI symptoms were the most common reason for readmission for allo-HCT, while neutropenic fever was the second most common for auto-HCT.

### Limitations

This study has several limitations. All findings are national estimates generated from the NRD sample; the accuracy of the estimates depends on the weighting method generated by AHRQ. The NRD is an administrative database and information on conditioning chemotherapy, other treatments received during the transplant, and disease burden and risk information are not available. The database is not positioned to assess social determinants of health, outpatient care, or other factors that influence patients’ health during transitions from hospital to home. The NRD administrative data used in this study are not designed to distinguish preventable readmissions. Readmissions across states are not captured by NRD; however, this may be inconsequential because very few patients will be readmitted in a state other than the one in which the procedure was performed after HCT, and the results did not differ when nonresidents were excluded. The data do not take into account the outpatient transplants being performed in some centers. Likewise, the data cannot be used to assess the effect of readmission on the outcomes of these patients. Data on race are not available in the NRD, so racial disparity on readmission cannot be estimated. Given the variability in the number of HCT performed per year globally due to differences in population size and resource constraints, the results should be interpreted with caution.

## Conclusions

Using a large national readmission database, we report the 30-day unplanned readmission rates after HCT in the United States and highlight that certain factors present during index admissions for HCT are associated with high adjusted readmission rates. This study found an inverse association between hospital HCT volume and readmission and distinguished several other factors associated with hospital readmission. Infections with and without neutropenic fever remain the most common reason for readmission with both HCT modalities. Lower readmission rates at high-volume hospitals may substantially reduce health care expenditure. These data will help identify various factors that may contribute to readmission after HCT and guide policy makers to identify and optimize HCT outcomes across hospitals.

## Notes

Supplement. **eTable 1.** *ICD-9* Codes **eFigure.** Predicted Probability of 30-Day Readmission With Annual HCT Volume **eTable 2.** Elixhauser Comorbidity Index for Readmission **eTable 3.** Univariate Analysis of 30-Day Unplanned Readmission vs None After Autologous and Allogeneic HCT **eTable 4.** Readmission Characteristics
