# Supplementary material for: Factors Associated With Unplanned 30-Day Readmissions After Hematopoietic Cell Transplantation Among US Hospitals
Source: JAMA Netw Open. 2019 Jul 5;2(7):e196476. doi: 10.1001/jamanetworkopen.2019.6476 (PMC12124690; doi:10.1001/jamanetworkopen.2019.6476)
Supplement: Supplement. — eTable 1. ICD-9 Codes eFigure. Predicted Probability of 30-Day Readmission With Annual HCT Volume eTable 2. Elixhauser Comorbidity Index for Readmission eTable 3. Univariate Analysis of 30-Day Unplanned Readmission vs None After Autologous and Allogeneic HCT eTable 4. Readmission Characteristics [file jamanetwopen-e196476-s001.pdf]

Supplementary Online Content

Dhakal B, Giri S, Levin A, et al. Factors associated with unplanned 30-day readmissions after hematopoietic cell transplantation among US hospitals. *JAMA Netw Open*. 2019;2(7):e196476. doi:10.1001/jamanetworkopen.2019.6476

eTable 1. *ICD-9* Codes

eFigure. Predicted Probability of 30-Day Readmission With Annual HCT Volume

eTable 2. Elixhauser Comorbidity Index for Readmission

eTable 3. Univariate Analysis of 30-Day Unplanned Readmission vs None After Autologous and Allogeneic HCT

eTable 4. Readmission Characteristics

This supplementary material has been provided by the authors to give readers additional information about their work.

© 2019 Dhakal B et al. *JAMA Network Open.*

eTable 1. ICD-9 Codes

**Variables**
Autologous HCT: 41.01, 41.04, 41.07 and 41.09<br>Allogeneic HCT: 41.03, 41.05, 41.06 and 41.08<br>Peripheral Blood: 41.04, 41.07, 41.05 and 41.08<br>Bone marrow: 41.00, 41.01, 41.02 and 41.03<br>Cord Blood: 41.06<br>Total Body irradiation: 92.24, 92.26, 92.77, 92.29<br>Causes:<br>1. Fever: 780.61<br>2. Neutropenic fever: 288.00-288.09<br>3. Documented Infections: 001.xx-037.xx, 039.xx-135.xx, 487.1, 487.8, 490, 465.xx., 466.xx, 595.0, 595.9, 595.89, 681.xx-682.xx<br>4. Bacteremia: 38.xx, 790.7<br>5. Mucositis: 528.x<br>6. CHF exacerbation (heart failure): 428.0, 428.21, 428.31, 428.41<br>7. Acute renal failure: 584.8, 584.9<br>8. Cardiac arrhythmias: 427.31, 427.32, 427.41, 427.0, 427.1, 427.2, 427.89, 785.0, 426.0, 426.10, 426.11, 426.12, 426.13<br>9. Sepsis: 995.91-995.91<br>10. Septic shock: 785.52<br>11. Fungal infection: 112.0, 112.5, 112.89, 112.9, 484.6, 117.3, 118, 117.9<br>12. CMV infection: 078.5<br>13. Graft versus host disease: 279.50 (allo-HCT only)<br>14. Failure to thrive: 783.41<br>15. GI symptoms: (diarrhea- 787.91), (nausea- 787.03) and (vomiting- ICD 787)<br>16. Hypovolemia/dehydration: 276.51-276.52\<br>17. Line infection: 999.31-999.32 and 995.91

© 2019 Dhakal B et al. *JAMA Network Open.*

eFigure. Predicted Probability of 30-Day Readmission With Annual HCT Volume

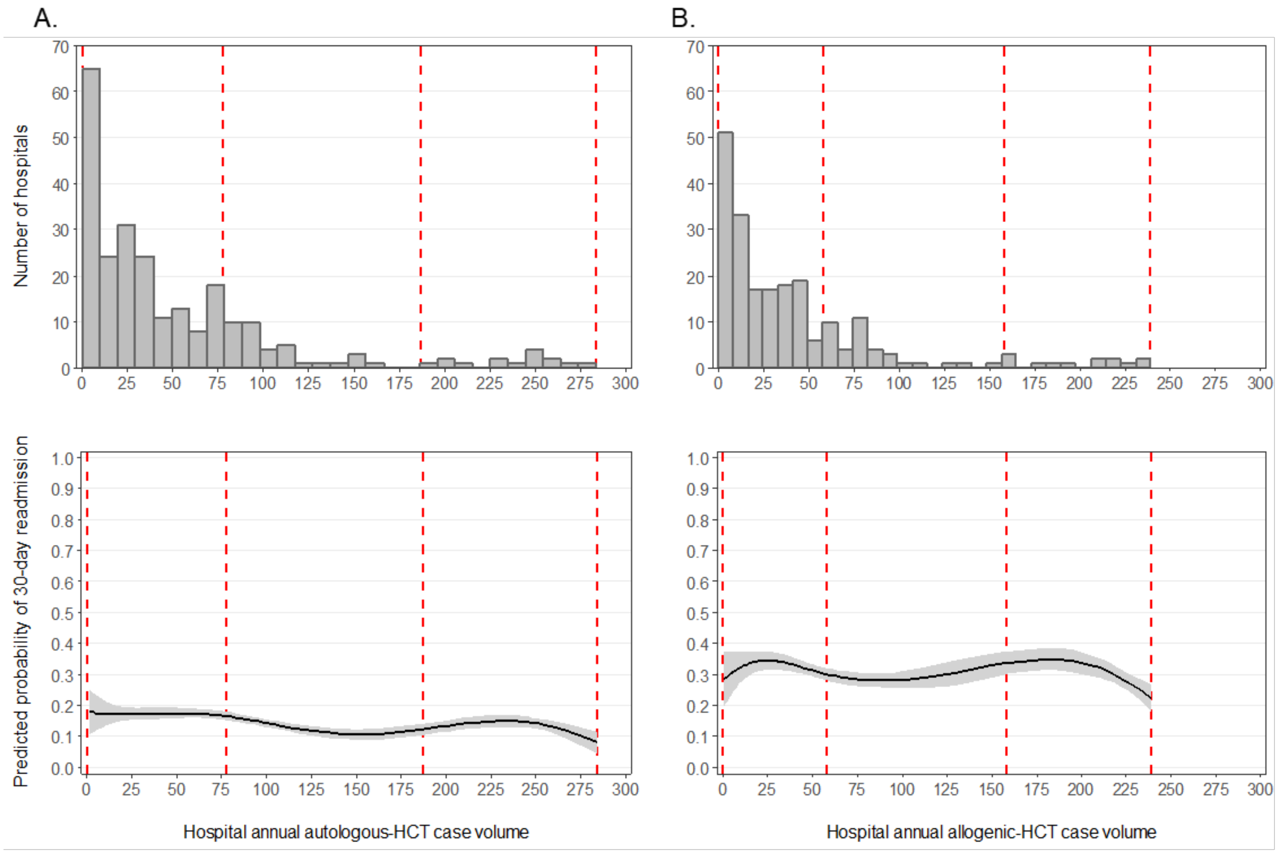

A) Predicted Probability of 30-Day Unplanned Readmission With Hospital Annual Autologous Transplant Volume.

B) Predicted Probability of 30-Day Unplanned Readmission With Hospital Annual Allogeneic Transplant Volume

© 2019 Dhakal B et al. *JAMA Network Open.*

eTable 2. Elixhauser Comorbidity Index for Readmission

**List of 29 Elixhauser co-morbidity measures used to calculate the readmission index**
1. Congestive heart failure<br>2. Valvular disease<br>3. Pulmonary circulation disorders<br>4. Peripheral vascular disease<br>5. Hypertension<br>6. Paralysis<br>7. Other neurological disorders<br>8. Chronic pulmonary disease<br>9. Diabetes without chronic complications<br>10. Diabetes with chronic complications<br>11. Hypothyroidism<br>12. Renal failure<br>13. Live disease<br>14. Chronic peptic ulcer disease<br>15. HIV and AIDS<br>16. Lymphoma<br>17. Metastatic cancer<br>18. Solid tumor without metastasis<br>19. Rheumatoid arthritis/collagen vascular diseases<br>20. Coagulation deficiency<br>21. Obesity<br>22. Weight loss<br>23. Fluid and electrolyte disorders<br>24. Blood loss anemia<br>25. Deficiency anemias<br>26. Alcohol abuse<br>27. Drug abuse<br>28. Psychoses<br>29. Depression

https://www.hcup-us.ahrq.gov/toolssoftware/comorbidityicd10/comorbidity_icd10.jsp

© 2019 Dhakal B et al. *JAMA Network Open.*

eTable 3. Univariate Analysis of 30-Day Unplanned Readmission vs None After Autologous and Allogeneic HCT

	**Autologous HCT (N = 28356)**			**Allogeneic HCT (N = 17213)**		
	**No 30-day readmission**	**30-day readmission**		**No 30-day readmission**	**30-day readmission**	
	**25038 (88.4%)**	**3318 (11.6%)**	**p-value**	**13012 (75.6%)**	**4201 (24.4%)**	**p-value**
**Hospital Volume**			0.01			0.04
Low	7823 (84.2%)	1467 (15.8%)		3990 (72.8%)	1493 (27.2%)	
Medium	8423 (90.1%)	922 (9.9%)		4321 (75.3%)	1421 (24.7%)	
High	8792 (90.4%)	929 (9.6%)		4700 (78.5%)	1287 (21.5%)	
**Year**			0.72			0.92
2012	8754 (89.4%)	1039 (10.6%)		3929 (75.4%)	1284 (24.6%)	
2013	8433 (87.4%)	1212 (12.6%)		4506 (76.2%)	1408 (23.8%)	
2014	7850 (88.0%)	1067 (12.0%)		4577 (75.2%)	1510 (24.8%)	
**Age**			0.007			0.05
18-49	5757 (86.3%)	911 (13.7%)		4816 (73.7%)	1723 (26.4%)	
>50	19282 (88.9%)	2407 (11.1%)		8195 (76.8%)	2478 (23.2%)	
**Sex**			0.10			0.87
Male	15284 (88.8%)	1935 (11.2%)		7595 (75.7%)	2441 (24.3%)	
Female	9754 (87.6%)	1383 (12.4%)		5417 (75.5%)	1761 (24.5%)	
**Stem cell source**			0.99			<0.001
Bone marrow	23840 (88.3%)	3159 (11.7%)		11351 (76.5%)	3495 (23.5%)	
Peripheral blood	1198 (88.3%)	159 (11.7%)		1308 (75.6%)	421 (24.4%)	
Cord Blood				353 (55.3%)	286 (44.8%)	
**Disease type**			0.12			0.01
MM	15040 (88.7%)	1921 (11.3%)		2072 (74.9%)	694 (25.1%)	
NHL	4950 (87.8%)	686 (12.2%)				
HL	1967 (90.0%)	218 (10.0%)				

© 2019 Dhakal B et al. *JAMA Network Open.*

AML				6120 (76.4%)	1895 (23.6%)	
ALL				1872 (70.9%)	768 (29.1%)	
MDS				1816 (77.6%)	524 (22.4%)	
Others	3081 (86.2%)	494 (13.8%)		1132 (78.0%)	320 (22.1%)	
**Insurance**			0.02			0.008
Medicare	7045 (88.0%)	964 (12.0%)		2549 (74.6%)	870 (25.5%)	
Medicaid	2597 (85.7%)	432 (14.3%)		1397 (71.6%)	555 (28.4%)	
Private/HMO	13979 (88.7%)	1791 (11.4%)		8273 (77.0%)	2468 (23.0%)	
Others	1418 (91.5%)	131 (8.5 %)		793 (72.0%)	308 (28.0%)	
**Median income quartile**			0.13			0.18
Lowest	4945 (86.3%)	783 (13.7%)		2191 (73.5%)	791 (26.5%)	
Lowest-Medium	6409 (88.6%)	824 (11.4%)		3033 (74.5%)	1040 (25.5%)	
Medium-High	6436 (89.1%)	787 (10.9%)		3533 (77.5%)	1027 (22.5%)	
Highest	6831 (88.9%)	855 (11.1%)		3986 (75.8%)	1274 (24.2%)	
Unknown	418 (85.9%)	68 (14.1%)		269 (79.3%)	70 (20.7%)	
**Elixhauser readmission index**			0.008			<0.001
≤ 0	6094 (88.8%)	767 (11.2%)		4017 (79.3%)	1051 (20.7%)	
1-9	7805 (88.8%)	984 (11.2%)		4242 (75.7%)	1361 (24.3%)	
10-19	6230 (89.7%)	713 (10.3%)		3006 (72.4%)	1148 (27.6%)	
≥ 20	4909 (85.2%)	854 (14.8%)		1747 (73.2%)	641 (26.9%)	
**Hospital Bed-size**			0.25			0.14
Small/Medium	4835 (90.4%)	513 (9.6%)		2983 (78.4%)	821 (21.6%)	
Large	20203 (87.8%)	2805 (12.2%)		10029 (74.8%)	3380 (25.2%)	
**Discharge disposition**			0.19			0.85
Routine	21371 (88.6%)	2763 (11.5%)		9380 (75.7%)	3016 (24.3%)	
Non-routine	3667 (86.9%)	555 (13.1%)		3632 (75.4%)	1186 (24.6%)	
**GVHD**			0.16			0.08
No	24862 (88.3%)	3282 (11.7 %)		11350 (76.0%)	3579 (24.0%)	

© 2019 Dhakal B et al. *JAMA Network Open.*

Yes	176 (82.9%)	36 (17.1%)		1662 (72.7%)	623 (27.3%)	
**Infections**			0.01			0.05
No	15461 (87.2%)	2266 (12.8%)		6726 (77.0%)	2010 (23.0%)	
Yes	9578 (90.1%)	1052 (9.9%)		6286 (74.1%)	2192 (25.9%)	
**Total body irradiation**			0.09			0.14
No	24898 (88.3%)	3287 (11.7%)		10972 (76.2%)	3436 (23.9%)	
Yes	141 (82.2%)	30 (17.8%)		2040 (72.7%)	765 (27.3%)	
**Index hospital LOS (days)**	17.7 [15.2, 20.6]	16.6 [7.8, 20.7]	< 0.001	24.6 [21.1, 30.2]	25.7 [21.3, 32.9]	0.89
**Index hospitalization cost ($1000s)**	49.8 [36.3, 75.1]	45.2 [28.2, 72.6]	0.005	94.1 [63.6, 149.1]	100.1 [68.3, 168.4]	0.06
Missing	3					

Frequencies of categorical variables are expressed as weighted N (row percent). Continuous variables are expressed as median and interquartile range, [25th percentile, 75th percentile].

© 2019 Dhakal B et al. *JAMA Network Open.*

eTable 4. Readmission Characteristics

	**Readmitted Autologous HCT**				**Readmitted Allogeneic HCT**			
	**Low**	**Medium**	**High**		**Low**	**Medium**	**High**	
	**(N = 1707)**	**(N = 1227)**	**(N = 1311)**	**p-value**	**(N = 1801)**	**(N = 1648)**	**(N = 1943)**	**p-value**
**Readmission type**				0.09				< 0.001
Non-elective	1467 (86%)	900 (75%)	929 (71%)		1478 (83%)	1421 (86%)	1287 (66%)	
Elective	240 (14%)	305 (25%)	382 (29%)		307 (17%)	227 (14%)	656 (34%)	
Missing		22			16			
**Readmission hospital**				0.26				0.28
Primary	1585 (93%)	1117 (91%)	1170 (89%)		1732 (96%)	1592 (97%)	1912 (98%)	
Different	122 (7%)	110 (9%)	141 (11%)		69 (4%)	56 (3%)	31 (2%)	
**Days to readmission**	5.4 [2.7, 10.4]	7.4 [3.6, 15.5]	5.9 [2.9, 12.4]	0.006	8.9 [4.6, 16.7]	9.9 [4.8, 17.1]	8.9 [3.9, 17.1]	0.32
**Readmission LOS (days)**	5.1 [2.7, 8.5]	4.7 [2.4, 10.5]	4.2 [2.3, 7.6]	0.24	6.9 [3.2, 15.2]	5.4 [2.7, 12.6]	5.3 [2.7, 11.7]	0.03
**Readmission costs ($1000s)**	12.9 [7.2, 23.7]	12.1 [6.8, 26.5]	12.1 [5.9, 22.2]	0.82	21.8 [10.0, 51.4]	19.0 [8.5, 45.7]	15.8 [8.3, 34.6]	0.22
Missing			4			1		

Frequencies of categorical variables are expressed as weighted N (column percent). Continuous variables are expressed as median and interquartile range, [25th percentile, 75th percentile].

© 2019 Dhakal B et al. *JAMA Network Open.*
